# Effects of *Nasturtium officinale* Extract on Antioxidant and Biochemical Parameters in Hemodialysis Patients: A Randomized Double-Blind Clinical Trial

**DOI:** 10.1155/2021/1632957

**Published:** 2021-01-08

**Authors:** Moslem Sedaghattalab, Marzieh Razazan, Hossein Sadeghi, Amir Hossein Doustimotlagh, Mehdi Akbartabar Toori, Rozina Abbasi Larki, Nahid Azarmehr, Arash Asfaram, Esmaeel Panahi kokhdan, Tahere Taheri, Aminollah Pourshohod

**Affiliations:** ^1^Department of Internal Medicine, Yasuj University of Medical Sciences, Yasuj, Iran; ^2^Medicinal Plants Research Center, Yasuj University of Medical Sciences, Yasuj, Iran; ^3^Department of Clinical Biochemistry, Faculty of Medicine, Yasuj University of Medical Sciences, Yasuj, Iran; ^4^Social Determinants of Health Research Center, School of Health and Nutrition Sciences, Yasuj University of Medical Sciences, Yasuj, Iran; ^5^Department of Nephrology, Yasuj University of Medical Sciences, Yasuj, Iran; ^6^Student Research Committee, Yasuj University of Medical Sciences, Yasuj, Iran; ^7^Medical-Surgical Nursing Department, Nursing Faculty, Yasuj University of Medical Science, Yasuj, Iran

## Abstract

**Background:**

Increased oxidative stress play an important role in the risk of cardiovascular disease, mortality, and mortality patients undergoing dialysis. *Nasturtium officinale *(watercress) contains numerous phytochemical compounds that act as an antioxidant by preventing oxidative damage to biomolecules. Therefore, this research aimed to explore the effect of the ethanolic extract of *Nasturtium officinale* (EENO) on antioxidant and biochemical markers of hemodialysis patients.

**Methods:**

In this double-blind, placebo-controlled trial, 46 hemodialysis patients were randomly recruited to consume either 500 mg/day EENO (*n* = 23) or placebo capsule (*n* = 23) for 4 weeks, at Shahid Beheshti Hospital, Yasuj, Iran, in 2019. Biomarkers of oxidative stress including glutathione peroxidase (GPX), superoxide dismutase (SOD), malondialdehyde (MDA), total oxidant status (TOS), total antioxidant capacity (TAC), and total sulfhydryl protein (T-SH) and biochemical parameters such as BUN, Hb, WBC, PLT, Ca, Ph, K, ALB, TChol, TG, LDL, and HDL were evaluated on days 0 and 28.

**Results:**

The serum levels of MDA and BUN significantly decreased after taking EENO supplementation (*P* < 0.001); however, SOD activity increased during the same period (*P* < 0.001). The serum levels of TAC remained constant in the intervention group, while it significantly declined in the placebo group (*P* < 0.09). The extract also prevented elevation in the serum levels of LDL and TG compared to the placebo group, although it was not statistically significant.

**Conclusions:**

The data indicated that the consumption of EENO improved some of the antioxidant parameters and minimizes the change in TG and LDL in hemodialysis patients. Therefore, due to the role of these factors in mortality and morbidity of dialysis patients, EENO can improve the condition of dialysis patients. However, more studies with longer intervention times and different doses of EENO are recommended.

## 1. Introduction

Chronic kidney disease (CKD) is a clinical syndrome specified by the progressive, slow, and permanent loss of kidney function. This process leads to catabolism (uremic toxins accumulation), altered electrolyte-water balance, and acid-base balance. It damages the physiological and biochemical functions of all other organs. Hemodialysis is the main therapy in the management of CKD, which can destroy amino acids, water-soluble peptides, and vitamins [1].

Oxidative stress and inflammation are some of the prominent features of CKD which are worsened by dialysis. These mediators can exacerbate kidney disease and many other complications in patients with CKD such as cardiovascular complications, malnutrition, and anemia [2]. The rates of cardiovascular problems in hemodialysis patients are 3–45 times higher than in the normal population, and almost 50% of these patients' deaths are due to cardiovascular disease. Even though the main risk factors for cardiovascular problems such as lipid disorders, diabetes, and hypertension have a high incidence in hemodialysis individuals, it cannot clarify the high prevalence and incidence of prior cardiovascular problems in hemodialysis patients [3]. Increased oxidative stress in these patients has several major causes; one of them is the loss of antioxidants during dialysis and the interaction between the blood and dialysis membrane [4]. Another reason is the bacterial products in dialysis, which directly or indirectly stimulate the secretion of free radicals by neutrophils. The third major cause is malnutrition in these patients, which reduces the absorption of dietary antioxidants. Besides, hemodialysis stimulates the immune cells and increases the production of reactive oxygen species, leading to an acute inflammatory reaction and oxidative stress [5]. All of these factors play important roles in the pathogenesis of atherosclerotic CVD. As a result, therapeutic strategies that decrease the production of reactive oxygen species can reduce the morbidity and mortality of patients with CKD [6]. Furthermore, the diagnostic value of some antioxidant parameters such as total FRAP (ferric ion reducing antioxidant parameter) and uric acid- (UA-) independent FRAP (FRAP-UA) has been reported for monitoring of CKD progression in children [7].

Recent research studies have shown the positive therapeutic effects of antioxidants on some biochemical parameters in the patient under hemodialysis [8]. *Nasturtium officinale* R. Br. (Watercress), belonging to Brassicaceae family, is a valuable source of various vitamins especially A, B, and C, folic acid, iodine, iron, protein, and calcium [9]. In traditional Iranian medicine, watercress is used to treat diabetes, bronchitis, wound, tuberculosis, influenza, and asthma [10]. Previous studies have demonstrated antioxidant [11–13], anti-inflammatory [14], hepatoprotective [15], nephroprotective [16], and anti-hyperlipidemia [17] properties of the watercress in vitro and in vivo conditions. Furthermore, it has been reported that watercress supplementation can decrease DNA injury to lymphocytes and modify blood antioxidant status in healthy subjects [18]. Consumption of watercress has a high ability to act as a source of anticancer drugs [19]. Watercress was found to contain several pigments, phenolic, and flavonoid compounds that antioxidant effects have been reported in various studies [20].

On the finding of previous studies, this placebo-controlled clinical trial was designed to assess the effect of ethanolic extract of Nasturtium officinal (EENO) supplementation on biochemical parameters and some biomarkers of oxidative stress, including the Glutathione peroxidase (GPX), superoxide dismutase (SOD), malondialdehyde (MDA), total oxidant status (TOS), total antioxidant capacity (TAC), and total sulfhydryl protein (T-SH) levels in hemodialysis patients.

## 2. Materials and Methods

### 2.1. Chemical Material

All solutions and buffers were provided using high purity chemical and enzymatic compounds (Sigma, USA). All colorimetric kits used in this investigation were purchased from ZellBio GmbH, Ulm, Germany.

### 2.2. Plant Collection

Aerial parts of *Nasturtium officinale* were collected from the rural part of Yasuj, Iran, in March–May 2019 and identified by the Department of Biology, Faculty of Sciences, Yasuj University of Medical Sciences. The collected herbal samples (herbarium no. HYU30230) were dried in a dark room at an appropriate temperature.

### 2.3. Preparation of Hydroalcoholic Extract

After collecting *Nasturtium officinale*, 1000 g of the plant powder is soaked in 5000 ml of ethanol 70% and kept at room temperature for 42 hours. After the initial extraction, the residue is extracted again by the solvent after 24 hours and added to the initial extract. The solvent is then evaporated at 40°C by a rotary and incubated at 37°C for a dry extract [14]. Components of the hydroalcoholic extract of *Nasturtium officinale* have been mentioned in our previous study.

### 2.4. Study Design

**Figure 1:**
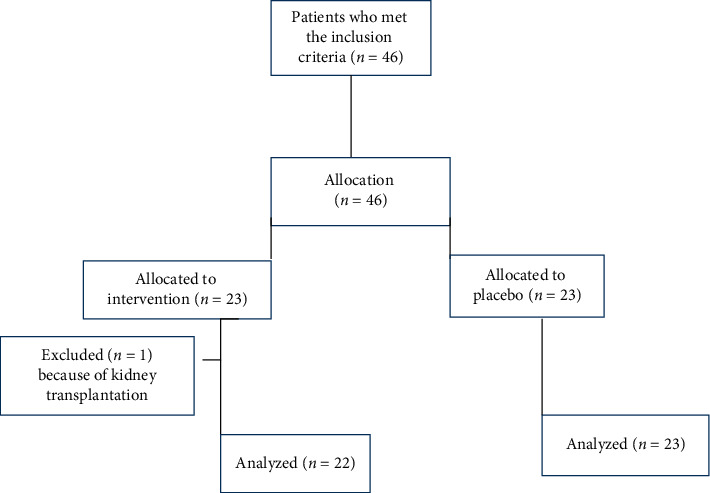
Flowchart of the study.

The current study was a double-blind randomized placebo-controlled trial to evaluate the effect of EENO on blood factors, lipid profile, and oxidative stress markers in hemodialysis patients who were referred to the nephrology clinic of Shahid Beheshti Hospital in Yasuj, Iran, 2019 (Figure 1). According to the inclusion criteria, 46 subjects were selected to participate in the study. The patients were randomly allocated into two groups (*n* = 23 patients per group) using quadruple blocks by block randomization.

Inclusion criteria were as follows: patients on maintenance dialysis for 3 months and two times a week, age ≥18 years, not taking medications such as corticosteroids, nonsteroidal anti-inflammatory drugs (NSAIDs), and additional complementary therapies with anti-inflammatory and antioxidant properties, without severe liver disorders, infectious diseases, the history of allergies to herbal supplements, or unpredictable drug side effects.

Subjects were excluded from the study if they received EENO supplementation less than 4 weeks; patients with preexisting cardiac arrhythmias, having hypotension, immunodeficiency, malnutrition and cachexia (BMI <18.5 kg/m^2^), albumin ≤3 mg/dl, and kidney transplantation were also excluded.

In the present study, patients and the researcher were not aware of the allocation of individuals in the two intervention or control groups, and the coding program was performed by a third person (who was not aware of the research).

### 2.5. Ethical Issue

The present research was conducted in line with the Helsinki principles. The procedure was accepted by Yasuj University of Medical Sciences' Ethics Committee (ethics code: YUMS.REC.1398.037); then, it was registered in the Iranian Clinical Trial System (http://www.irct.ir) with the registration number of IRCT20150622022869N7. Before participating in the study, informed consent was received from all individuals, and they were told of their right to withdraw from the research at any time.

### 2.6. Trial Interventions

The participants were assigned to take a capsule that contained 500 mg of EENO or a similar placebo capsule once a day for 4 weeks. The placebo capsule contained wheat flour. To ensure the patient took the supplement, they were asked to bring the capsule container to the next dialysis session. Patients were advised to avoid consuming other herbal and antioxidant supplements to maintain their normal eating habits, lifestyle, and physical activity during the study. Participants took the supplementation and placebo in identical in terms of color, shape, packaging, and size.

The dose of *Nasturtium officinale* was selected based on conversion of animal's dose to human [21]. In animal studies, a dose of 50 mg/kg was able to show nephroprotective and antioxidant effects against gentamycin-induced nephrotoxicity in rats, so this dose was converted to human dose [22].

Anthropometric factors, including bodyweight and BMI, were collected both at the baseline and four weeks after the study. The etiology of end-stage renal failure was chronic hypertension (*n* = 18), diabetic nephropathy (*n* = 22), polycystic kidney disease (*n* = 2), and other disorders (*n* = 4).

### 2.7. Blood Samples

Blood samples (10 cc, before the dialysis) were taken two times: at the beginning and the end of the trial. Isolated serum kept at −20°C for evaluation of biochemical markers [23] such as blood urea nitrogen (BUN), hemoglobin (Hb), white blood cell (WBC), platelet (PLT), calcium (Ca), phosphor (Ph), potassium (K), albumin (ALB), triglyceride (TG), total cholesterol (TChol), high-density lipoprotein (HDL), and low-density lipoprotein (LDL). Parameters related to stress oxidative such as glutathione peroxidase (GPX), superoxide dismutase (SOD), malondialdehyde (MDA), total antioxidant capacity (TAC), total oxidant status (TOS), and total sulfhydryl protein (T-SH) were also evaluated.

### 2.8. Biochemical Markers Measurements

Biochemical parameters (BUN, Hb, WBC, PLT, Ca, Ph, K, ALB, TChol, TG, LDL, and HDL) were measured via a standard automated analyzer using standard system commercial kits.

### 2.9. Measurement of Antioxidant Parameters

Plasma malondialdehyde (MDA) levels were assessed using the TBA reaction assay and expressed as *µ*mol/ml [24].

Total thiols (T-SH) level was measured based on reaction with DTNB [25]. Glutathione peroxidase (GPx), superoxide dismutase (SOD), total oxidant status (TOS), and total antioxidant capacity (TAC) activity were determined by colorimetric kits (ZellBio GmbH, Ulm, Germany) using a spectrophotometer [15, 26–28].

The intraassay CVs for GPX, SOD, TOS, and TAC were 3.5%, 5.8%, less than 4.2%, and less than 3.4%, respectively. The interassay CVs for GPX, SOD, TOS, and TAC were 4.7%, 7.2%, less than 6.9%, and less than 4.2%, respectively. The lower limit of detection of GPX, SOD, TOS, and TAC were 5 U/mL, 1 U/mL, 0.5 *µ*M, and 0.1 mM, respectively.

### 2.10. Statistical Analyses

Considering MDA as a key variable and the work of Mazani and colleagues [29], the sample size was calculated with *α* = 0.05, 90% power to following formula.

(1) $$\begin{array}{c} n=\frac{{\left(Z_{1}-\left(\alpha /2\right)+Z_{1}-\beta \right)}^{2}\left(\delta_{1}^{2}+\delta_{2}^{2}\right)}{{\left(\mu_{1}-\mu_{2}\right)}^{2}}. \end{array}$$

The calculated sample size was 17 patients in each group; however, to avoid losing sample during study, 23 subjects were considered for each group.

The data were analyzed by SPSS 16 software. Parametric tests were used for normal distribution, and nonparametric tests were used for data without normal distribution. Quantitative data analysis was performed using the Kolmogorov–Smirnov test. Paired sample *t*-tests and Wilcoxon signed ranks test were used to evaluate the differences within each group before and after intervention, respectively, with normal distribution and without normal distribution. To compare the difference between pre and post-intervention in the intervention and control groups, the independent sample *t*-test was used for normal distribution, and the Mann–Whitney *U* test was used for data without normal distribution [30].

## 3. Results

### 3.1. Basic Characteristics of the Patients

46 patients who met the inclusion criteria for this study were enrolled in the study and randomized into two groups. Figure 1 provides a flowchart of the clinical analysis. One subject from the intervention group was withdrawn because of kidney transplantation.

Data analysis was performed on 22 EENO patients and 23 placebo patients.

Tables 1 and 2 illustrate the basic demographic data of the patient. The average age of the placebo group and intervention group was 63.1 ± 13 and 58.9 ± 16 years, respectively. The mean BMI was 23.8 ± 4.2 in the control group and 25.6 ± 4.2 in the intervention group (*P* value >0.05). 40% of the control group and 59% of the intervention group had diabetes. There was no substantial variance in the two groups in terms of the basic features.

Forty-five participants completed the study (23 placebo groups, 22 EENO groups). One subject from the intervention group was withdrawn because of kidney transplantation.

### 3.2. Effect of EENO on Biochemical Parameters

Table 3 presented the biochemical parameters of the participants at the baseline and after the intervention (4 weeks) in each group. There was no statistically significant difference in the mean serum of WBC, Hb, Plt, K, Ca, P, and Alb levels between intervention and control groups. EENO supplementation reduced total cholesterol, LDL, and TG, as well as increased HDL. However, these changes were not statistically significant compared to placebo individuals.

A significant decrease was observed in the BUN levels in the EENO grouped compared with placebo subjects (40.6 ± 11.2 vs. 34.6 ± 15.1, *P* < 0.04). In the placebo group, BUN slightly increased during the study. The net difference in serum BUN levels of the control group at the baseline and the end of the study was 2.04 ± 14.3 and −6 ± 12.5 in the placebo and intervention groups, respectively.

### 3.3. Effect of EENO on Antioxidant Parameters

No significant alteration was observed in the values of MDA, SOD, T-SH, TAC, GPX, and TOS between groups (Table 4). Analysis of the data showed that the consumption of EENO induced a significant improvement in some markers of antioxidant during 4 weeks (Table 1).

At the beginning of the study, there was no statistical difference between the control and intervention groups in terms of the MDA level, but at the end of the investigation, a statistically significant reduction was observed in both intervention (500 mg/day) and placebo groups at the MDA level (*P* < 0.001). The net reduction in the MDA level in the EENO supplementation group (−1.1 ± 0.28 mg/dL) was significantly greater than that observed in the placebo group (−0.86 ± 0.26 mg/dL) (*P* < 0.003).

As presented in Table 4, SOD activity significantly increased in both EENO (500 mg/day) and placebo groups compared to the baseline value (*P* < 0.001), even though the net increase in the EENO group (7.7 ± 10) was more than placebo individuals (5.5 ± 4.9).

Treatment with EENO supplementation (500 mg/day) for 4 weeks did not affect the activity of GPx (Table 4).

As presented in Table 4, T-SH levels in the placebo and EENO groups (500 mg/day) significantly reduced compared to the baseline levels (*P* < 0.001).

TAC levels were remaining constant in patients who received EENO (500 mg/day), for 4 weeks compared to the baseline value, although the reduction in the TAC value was obvious in the placebo group (*P* < 0.09) compared to the baseline levels.

TOS value significantly decreased in both EENO (500 mg/day) and placebo groups compared to the baseline value (*P* < 0.001); however, the decrease in the EENO individuals (−4.4 ± 4.2) was more than that observed in the placebo group (−3.6 ± 3.3).

## 4. Discussion

The present study provided some evidence for the first time that a 4-week supplement with EENO (500 mg/day) was associated with a significant decrease in the serum levels of BUN and MDA and rise in SOD values.

It is well known that oxidative stress surge in HD patients and play an important role in cardiovascular disease, morbidity, and mortality in these patients [31, 32]. MDA is one of the end products of peroxidation of unsaturated fatty acids in the cells of living organisms. Increased generation of free radicals causes overproduction of MDA. Therefore, MDA, as a marker of lipid peroxidation, is widely used as an assay for evaluating oxidative stress [15]. Our result showed that the consumption of 500 mg/day of EENO significantly reduced the serum level of MDA compared to baseline and placebo groups. This finding is in good agreement with a study, which reported that EENO reduced MDA levels in people with physical disabilities [33]. In this line, it has been reported that ethanolic extract of *Nasturtium officinale* was able to reduce cellular lipid peroxidation and scavenger of superoxide anion radical [33]. Yazdanparast and their colleagues confirmed that EENO inhibited the extent of lipid peroxidation in the liver of hypercholesteremic rats [34]. Besides, EENO reduced the serum level of MDA and tissue damage in some animal laboratory models such as renal disease, liver injury, diabetes, and inflammation [14, 15, 26, 35].

SOD as an antioxidant enzyme is existent in extracellular fluids and almost all aerobic cells. This enzyme is the first and very important line of defense enzymes against oxidative stress, especially superoxide anion radicals [36]. The data from this present study revealed that the SOD value increased in both EENO supplementation and placebo groups compared to the baseline value, but the increase in the EENO group was greater than that observed in placebo individuals. These findings confirm the previous results that showed EENO increased the SOD activity in hypercholesterolaemic rats [34]. Our results also are consistent with the previous study that reported EENO increased the activity of SOD in diabetic rats [35]. Contrary to the results of the present study, Clemente and coworkers, in a human clinical trial on the antioxidants effect of EENO supplementation in people with a physical disability, showed that EENO fails to increase the SOD activity [33].

GPx, as a cytosolic enzyme, catalyzes the reduction of hydrogen peroxide to water and oxygen, as well as peroxide radicals to alcohols and oxygen. Our results showed that EENO supplementation in the intervention group did not affect the activity of this enzyme. Contrary to the finding of the current work, in some animal models of hypercholesterolemia and diabetes in rats, EENO increased the activity of GPx [34, 35]. This difference can be due to the difference between animal models with the clinical trials.

Thiol groups are key members of antioxidants that have a vital function in the scavenger of free radicals by enzymatic and nonenzymatic mechanisms [26]. The total thiol group of proteins is primarily responsible for their antioxidant activity; therefore, they can evaluate as an indicator of oxidative stress [37]. Our data showed that T-SH levels in the placebo and intervention groups were significantly reduced. In contrast to the results, some studies showed that EENO increased the level of T-SH both in acetaminophen-induced liver toxicity and bile duct ligation-induced liver fibrosis in animal models [15, 26]. Besides, a study reported that EENO increased the T-SH level in the cyclophosphamide-induced hepatotoxicity in rats [38].

Due to the cumulative effects of all antioxidants in plasma and body fluid, total antioxidant capacity can provide more important scientific evidence than the evaluation of single biomarkers. An improvement in plasma antioxidant potential indicates the rate of antioxidant uptake and high antioxidant status in the body or the activation of a regulatory process in free radicals [39]. Senol et al. observed that patients with hemodialysis have a higher concentration of serum urea and total antioxidant potential compared to healthy volunteers [40]. The results of the current study showed that the TAC value remains constant in patients who received EENO compared to the baseline value, although the TAC value in the placebo group decreased significantly compared to the baseline value. Also, TOS levels decreased in both groups compared to the baseline value; however, the reduction was greater in the EENO subjects than in the placebo group.

Overall, these findings could indicate the antioxidant effects of EENO supplementation on dialysis patients. The presence of high content of phenolics and flavonoids in EENO has been confirmed in several previous studies [41]. In this line, some EENO ingredients such as glucosinolate, chlorogenic, and caffeic acid have robust antioxidant and anti-inflammatory activities [42]. Therefore, high phenolic and flavonoid EENO components may be responsible for the antioxidant effects observed in the present study [43].

In our investigation, the mean of BUN in the supplementation individuals decreased compared to baseline levels, and this change was significant compared with the placebo group. In accordance with our investigation, Karami et al. showed that administering EENO attenuated vancomycin-induced nephrotoxicity and reduced BUN levels due to vancomycin injection [16]. Another study by Shahani et al. represented that the EENO extract inhibited the gentamicin-induced toxicity in rats and decreased BUN and inflammatory parameters in rats [22]. Urea is commonly considering as a nontoxic substance in body, but it can convert to highly toxic cyanides that bind and alter the structure proteins by carbamylation including serum albumin. It has been shown that carbamylated serum albumin is a risk factor for in subjects with kidney failure. Hence, it is may be valuable to adjust urea levels in these patients with medication or supplementation. Indeed, in the present study, it observed a significant decrease in the serum level of BUN in patients that received EENO supplementation.

The results of the present study showed that the TG and LDL values had a significant increase in the placebo group compared to the baseline levels, but in the intervention individuals, the blood lipid levels were kept constant compared to the baseline level. Therefore, it seems that the plant extract was able to prevent an increase in blood lipid levels in hemodialysis patients. In this regard, it has been reported that EENO dramatically reduced the TG and LDL and raised HDL in high-fat diet rats [34]. In addition, it has been reported that treatment with EENO at a dose of 200 mg/kg showed a significant hypolipidemic effect in diabetic rats [44].

This study had the following limitations: the condition of patients and treatment plan did not allow us to increase the duration of the intervention by more than 4 weeks. Also, due to the treatment protocol of the patients, we could not prescribe more than 500 mg/day of the extract per day. Therefore, the main limitations of the present study were short duration of treatment and dosage of EENO supplement.

## 5. Conclusions

In general, a 4-week consumption of EENO was well tolerated in hemodialysis patients. The present results showed that EENO improved some parameters of serum antioxidants and minimized the change in serum levels of TG and LDL in dialysis patients. Therefore, due to the role of these factors in mortality and morbidity of dialysis patients, it is suggested that EENO supplementation can improve the condition of hemodialysis patients, although further clinical trials with diverse doses of EENO and longer intervention periods are recommended to evaluate these favorable effects. We also suggest more studies to evaluate the potential benefits of EENO in other types of kidney disorders such as individuals with chronic kidney disorder or in the early phases of kidney failure.

## Figures and Tables

**Table 1:** Basic characteristics of hemodialysis patients in the EENO and the placebo groups.

	Groups	Mean ± SD	*P* value
Age	Control	63.1 ± 13	*P* < 0.36
	EENO	58.9 ± 16	

BMI	Control	23.8 ± 4.2	*P* < 0.16
	EENO	25.6 ± 4.2	

**Table 2:** Chi-square test of independence of the relationship between diabetic patients for both control and intervention groups in the study of the effect of hydroalcoholic extract of aerial parts of *Nasturtium officinale* on antioxidant and biochemical parameters of chronic hemodialysis patients: a randomized double-blind clinical trial.

Levels	Group		Chi-square	*P* value
	Control	Intervention		
Number of patients	9	13	1.39	0.238
Patients with diabetes (%)	40%	59%		

**Table 3:** Comparison of differences in biochemical parameters during the study between the control and EENO groups.

Variable		Baseline	After 4 weeks	*P* value	Net differences of groups	*P* value
BUN (mg/dl)	Control	39.8 ± 9.4	42 ± 11.6	*P* < 0.5	2.04 ± 14.3	*P* < 0.05^*∗*^
	Intervention	40.6 ± 11.2	34.6 ± 15.1	*P* < 0.04^*∗*^	−6 ± 12.5	
WBC	Control	5474 ± 1737.1	5339 ± 1567	*P* < 0.93	−134.8 ± 1491.7	*P* < 0.5
	Intervention	6308.7 ± 926.1	6465 ± 2209	*P* < 0.47	156.5 ± 995.3	
Hb (g/dl)	Control	10.7 ± 1.42	11.2 ± 1.56	*P* < 0.04^*∗*^	0.58 ± 1.2	*P* < 0.6
	Intervention	10.4 ± 1.47	11.2 ± 1.7	*P* < 0.02	0.79 ± 1.5	
Plt	Control	153695 ± 48760.5	153956.5 ± 44604.8	*P* < 0.97	260.9 ± 41377.6	*P* < 0.2
	Intervention	178565.2 ± 59549.7	158260.9 ± 45508.5	*P* < 0.2	−20304 ± 48280.8	
K (mmol/L)	Control	4.4 ± 0.35	4.6 ± 0.61	*P* < 0.27	0.13 ± 0.58	*P* < 0.6
	Intervention	4.6 ± 0.53	4.6 ± 0.73	*P* < 0.84	0.02 ± 0.59	
Ca (mg/dl)	Control	9.4 ± 1.13	10.7 ± 1.8	*P* < 0.005^*∗∗*^	1.3 ± 2	*P* < 0.7
	Intervention	8.8 ± 1.32	10.4 ± 2	*P* < 0.001^*∗∗∗*^	1.5 ± 1.6	
Ph (mg/dl)	Control	4.5 ± 1	3.7 ± 1	*P* < 0.93	−0.8 ± 1.6	*P* < 0.6
	Intervention	4.8 ± 0.6	4 ± 1.3	*P* < 0.47	−0.8 ± 1.5	
Alb (g/dl)	Control	3.9 ± 0.4	3.9 ± 0.3	*P* < 0.72	0.01 ± 0.406	*P* < 0.5
	Intervention	3.8 ± 0.43	3.7 ± 0.4	*P* < 0.71	−0.07 ± 0.456	
TG (mg/dl)	Control	91.4 ± 59.6	115.3 ± 96.3	*P* < 0.01^*∗∗*^	23.9 ± 44.2	*P* < 0.34
	Intervention	101 ± 57.1	109.1 ± 51.8	*P* < 0.24	8.2 ± 38.7	
TChol (mg/dl)	Control	114 ± 27.4	117.7 ± 33	*P* < 0.42	3.65 ± 21.1	*P* < 0.62
	Intervention	130.2 ± 27.7	129.3 ± 32.6	*P* < 0.95	−0.91 ± 26.1	
LDL (mg/dl)	Control	51.6 ± 7.8	58.2 ± 20.8	*P* < 0.04^*∗*^	6.6 ± 14	*P* < 0.2
	Intervention	65 ± 22.5	64 ± 20.9	*P* < 0.77	−1.1 ± 17	
HDL (mg/dl)	Control	38 ± 12.3	38.5 ± 9.9	*P* < 0.64	0.52 ± 5.3	*P* < 0.31
	Intervention	37.7 ± 11	38.9 ± 11.3	*P* < 0.28	1.2 ± 5.3	

BUN, blood urea nitrogen; WBC, white blood cell; Hb, hemoglobin; Plt, platelet; TChol, total cholesterol; LDL, low-density lipoprotein; HDL, high-density lipoprotein; TG, triglycerides; K, potassium; Alb, albumin; Ca, calcium; P, phosphorus ^*∗*^(*P* < 0.05), ^*∗∗*^(*P* < 0.01), and ^*∗∗∗*^(*P* < 0.001).

**Table 4:** Comparison of changes in the antioxidant markers during the study period between the placebo and EENO groups.

Variable		Baseline	After 4 weeks	*P* value	Net differences of groups	*P* value
TAC (mM)	Control	0.72 ± 0.25	0.63 ± 0.3	*P* < 0.09	−0.09 ± 0.24	*P* < 0.39
	Intervention	0.7 ± 0.25	0.7 ± 0.3	*P* < 0.77	0.009 ± 0.29	
TOS (*µ*M)	Control	9.5 ± 2.3	5.9 ± 2.3	*P* < 0.001^*∗∗∗*^	−3.6 ± 3.3	*P* < 0.47
	Intervention	11.3 ± 3.3	6.9 ± 2.4	*P* < 0.001^*∗∗∗*^	−4.4 ± 4.2	
GPX (U/mL)	Control	223 ± 115	252.9 ± 107.8	*P* < 0.17	29.9 ± 7.8	*P* < 0.54
	Intervention	164.2 ± 103.6	173.3 ± 134.4	*P* < 0.56	9.1 ± 134.8	
T-SH (mmol/L)	Control	10.1 ± 4.1	5.6 ± 3.2	*P* < 0.001^*∗∗∗*^	−4.5 ± 5.5	*P* < 0.323
	Intervention	13.1 ± 5.3	7.4 ± 4.3	*P* < 0.001^*∗∗∗*^	−5.6 ± 5.5	
MDA (mmol/L)	Control	1.5 ± 0.14	0.67 ± 0.22	*P* < 0.001^*∗∗∗*^	−0.86 ± 0.26	*P* < 0.001^*∗∗*^
	Intervention	1.6 ± 0.13	0.42 ± 0.27	*P* < 0.001^*∗∗∗*^	−1.1 ± 0.28	
SOD (U/mL)	Control	28.3 ± 8.1	33.8 ± 5.7	*P* < 0.001^*∗∗∗*^	5.5 ± 4.9	*P* < 0.32
	Intervention	29.3 ± 6.3	37.1 ± 8.4	*P* < 0.001^*∗∗∗*^	7.8 ± 10	

TAC, total antioxidant capacity; TOS, total oxidant status; GPX, glutathione peroxidase; T-SH, sulfhydryl protein; MDA, malondialdehyde; SOD, superoxide dismutase ^*∗∗*^(*P* < 0.01) and ^*∗∗∗*^(*P* < 0.001).

## Data Availability

The data used to support the findings of this study are available from the corresponding author upon request.
